# Centralized Duplicate Removal Video Storage System with Privacy Preservation in IoT

**DOI:** 10.3390/s18061814

**Published:** 2018-06-04

**Authors:** Hongyang Yan, Xuan Li, Yu Wang, Chunfu Jia

**Affiliations:** 1 College of Computer and Control Engineering, Nankai University, Tianjin 300071, China; hyang.yan@foxmail.com (H.Y.); cfjia@nankai.edu.cn (C.J.); 2 College of Mathematics and Informatics, Fujian Normal University, Fuzhou 350117, China; 3 School of Computer Science, Guangzhou University, Guangzhou 510006, China; yuwang@gzhu.edu.cn

**Keywords:** Internet of Things, cloud storage environment, data deduplication, privacy preservation, cryptosystem

## Abstract

In recent years, the Internet of Things (IoT) has found wide application and attracted much attention. Since most of the end-terminals in IoT have limited capabilities for storage and computing, it has become a trend to outsource the data from local to cloud computing. To further reduce the communication bandwidth and storage space, data deduplication has been widely adopted to eliminate the redundant data. However, since data collected in IoT are sensitive and closely related to users’ personal information, the privacy protection of users’ information becomes a challenge. As the channels, like the wireless channels between the terminals and the cloud servers in IoT, are public and the cloud servers are not fully trusted, data have to be encrypted before being uploaded to the cloud. However, encryption makes the performance of deduplication by the cloud server difficult because the ciphertext will be different even if the underlying plaintext is identical. In this paper, we build a centralized privacy-preserving duplicate removal storage system, which supports both file-level and block-level deduplication. In order to avoid the leakage of statistical information of data, Intel Software Guard Extensions (SGX) technology is utilized to protect the deduplication process on the cloud server. The results of the experimental analysis demonstrate that the new scheme can significantly improve the deduplication efficiency and enhance the security. It is envisioned that the duplicated removal system with privacy preservation will be of great use in the centralized storage environment of IoT.

## 1. Introduction

In recent years, with the increase in the number of devices connecting to Internet, the concept of the “Internet of Things” (IoT) has gradually attracted much attention from a large number of researchers. The main idea of IoT is to make an ever increasing number of objects connected and to enable them to interact with each other. In this context, the role of smart devices has transformed from traditional communication tools to a modern role of acquisition, processing and communication [1,2,3,4].

Connecting and interactive intelligent objects in IoT include mobile devices, wearable sensor devices and environmental sensors. These objects can project a digital snapshot of their state, activity and the surroundings in common scenarios such as home, working office, and vehicles. The information is communicated among interconnected objects and provided to users. Once IoT is widely adopted, smart objects will densely populate our environment and help to enhance our digital experiences and interactions with our surroundings. For example, when people attach a wireless sensor to their pets, they will be able to check where their pets are in real-time. If a gas leak is detected in a building, the alarm will be automatically set off. When Google glasses are worn, the user can take photos, send messages and search for information on the internet simply with voice control. In addition, some applications in the IoT need to capture videos. For example, traffic and environmental monitoring devices in smart cities, or smart cameras in a smart home. Therefore, the context providers can deliver useful services to the users with the information gathered by smart terminals.

However, most of the devices in IoT have the disadvantage of a limited storage capacity and computing power. With the popularity of IoT applications, more and more data need to be collected and processed by content producers, which may lead to unaffordable storage space and computational costs for IoT terminals. Compared to increasing the capabilities of terminal devices, a more convenient way is to resort to the resource-rich cloud center. With the advent of the 5G era, data transmission on the Internet will be more efficient. In fact, people are getting used to transferring their data to remote servers for centralized management, so it is conceivable that the cloud platform will play an important role in the applications of IoT. The terminals can exploit the cloud platform to manage the data from the IoT due to the advantages of data sharing [5], access control [6,7,8,9], data authentication [10], data analysis [11], and verifiable computing in the cloud [12,13]. In this context, the devices in charge of gathering information at the terminals can be relieved from storing and processing the data. This leads to the inevitability of the combination of IoT and the cloud platform. Therefore, the applications in IoT can utilize the storage capability and computing power of the cloud platform to process the context provided by the terminals. Indeed, there are already web services that rely on the context provider in the cloud, such as weather services, online calendars and environmental sensors.

The large scale of data created by IoT devices leads to the consumption of communication bandwidth and storage space because there is a large amount of redundancy in the data. Thus, data deduplication has been widely adopted to eliminate the redundant data. Nevertheless, data collected in IoT are sensitive and closely related to users’ personal information; thus, the privacy protection of users’ information becomes a challenge. For example, security and privacy issues must be taken seriously when sensor data is transmitted from the peripheral to the central part of the structure. Take wireless technologies, for example, which are the current IoT forecast trends due to their mobility and service portability. The common wireless scenarios in the IoT include RFID and Wireless Sensor Networks. Because wireless devices share the physical medium with other devices when transmitting the sensor data to the center structure, including the potentially malicious devices, their extensive application obviously brings a great security challenge [14]. For example, when people use wearable intelligent equipment, they emphasize their worries about whether these devices with camera shooting and sound recording will invade their privacy. Thus, it is better to allow the users to control their own privacy data. The general solution [15,16] is that the devices should have two context-based operating modes: public and personal. Then, the user can choose the personal model and define the secure policy when they require privacy protection. Under the personal model, some devices can provide services, e.g., the communication of information gathered by the environment sensors, only with the objects that it has a close relationship with and that already possess the public key, such as objects belonging to the same user.

Therefore, it is necessary for data to be encrypted before being uploaded to the cloud. Data encryption is a practical privacy protection security method in this context. During the process of encrypted sensor data being transferred to the central structure from the peripheral device, even if other unauthorized devices, including potentially malicious devices, on the sharing wireless physical medium can intercept the information, they are not able to decrypt the data. This method can effectively reduce the risk of data disclosure, but encryption makes the performance of deduplication by the cloud server difficult because the ciphertext will be different even if the underlying plaintext is identical. This brings new challenges to the application of the IoT.

To address this problem, the general solution is the homomorphic encryption technique [17,18]. However, due to the problems of high computational complexity and low efficiency, homomorphic encryption is not suitable for high dimensional data removal, especially in the field of IoT. Thus, the emergence of Intel software guard extension (SGX) technology provides us with a new way to solve the above problem. SGX is a new CPU extension for executing secure code in Intel processors. It can provide a secure environment for program and data, any attackers cannot read or modify the data in it. Based on this, we can load the encrypted data into the secure environment, decrypt it, and then compute in plaintext form. This solution ensures not just the privacy of data, but makes data processing easier.

### 1.1. Related Work

The widespread use of IoT devices has led to the explosive growth of data. However, much research has pointed out that a large amount of data stored in the cloud is duplicated. Duplicate data takes up a lot of space and it brings a bottleneck to data storage. Data deduplication [19,20,21] has become an important technique to be adopted in storage systems, because it saves storage space and bandwidth for users. To protect data privacy, all the operations and computations must be conducted in encrypted form. Thus, both the efficiency and accuracy rate are reduced.

Currently, the deduplication methods can be divided to two categories according to chunk level: file-level deduplication [22,23,24] and block-level deduplication [25,26]. In the file-level method, by computing a hash value for the file, a file with the same hash value is only stored if each bit of it matches the original file exactly [27]. Other duplicated files are deleted. This method is easy to implement, but the deduplication effect is poor. If the file changes by one byte, the deduplication cannot be realized. In the block-level method, each file is firstly divided into several blocks and the deduplication is conducted on the blocks. This method is more effective than the first one, and it is suitable for deduplication of partial content change.

Besides the above works, there are some other related deduplication works, such as key management in secure deduplication [28], proof of ownership for deduplication [29,30], supporting privacy-preserving fuzzy deduplication [31], secure authorized deduplication [32], and distributed deduplication [33]. However, as far as we know, most of the current deduplication systems focus on the study of text and images, and only a few works [34,35,36] have researched video. Until now, there has not been a secure framework to address the problem of privacy-preserving video deduplication.

### 1.2. Our Contribution

In our view, there are only a few differences between continuous frames due to the correlation in the video data; thus, it is very necessary to perform deduplication on video data. In view of the importance of duplicate removal storage systems in IoT, this paper addresses the problem of privacy-preserving video deduplication on the servers of the cloud center. To summarize, we make the following contributions:

- We present a method of secure video deduplication with privacy preservation that enables users to remove the encrypted duplicated data with the help of the server. According to the chunk level, two secure systems are proposed: a file-level deduplication system and block-level deduplication system.
- We adopt symmetric encryption and convergent encryption in file-level and block-level deduplication systems to prevent statistical information leakage.
- We apply the SGX technique in the block-level deduplication system to guarantee privacy security.

The remainder of this paper is organized as follows. In Section 2, we describe some necessary preliminaries. In Section 3, we formally formulate the problem of centralized privacy-preserving image deduplication. In Section 4, we give the file-level and block-level deduplication schemes. Section 5 presents the experimental results. Finally, the conclusions and future work are given in Section 6.

## 2. Preliminaries

### 2.1. Symmetric Encryption

In cryptosystems, the original message, usually known as plaintext, is encrypted to ciphertext. It is incomprehensible if people do not have the correct decryption key. The encryption method can be divided into two schemes: symmetric encryption and asymmetric encryption. Here, we introduce the symmetric encryption which is adopted in the proposed scheme.

In symmetric encryption, the sender encrypts the plaintext message, *P*, with the secret key, *K*, to obtain the ciphertext, *C*, with the following equation:

(1) C=Sym_Enc(P,K)

If the receiver receives the ciphertext, *C*, he can decrypt it with the same key, *K*, to recover the plaintext, *P*:

(2) P=Sym_Dec(C,K)

Note that the key, *K*, should be shared securely between the sender and receiver. The key has to be kept secret from others.

### 2.2. Convergent Encryption

The convergent encryption [37,38] is a specific encryption method which generates the same key from the same plaintext. It guarantees different users get the same key if their plaintexts are same. Thus, the crucial point of convergent encryption is the generation of the key.

(3) K=Gen(P)

Then, the key can be used to encrypt the plaintext and output the ciphertext.

(4) C=Enc(P,K)

In this paper, convergent encryption is used to realize the private-preserving multimedia deduplication system.

### 2.3. Intel SGX Background

Intel Software Guard Extensions (SGX) [39,40] is a new CPU extension for executing secure code in Intel processors. During the execution, it allows for the creation of isolated execution environments which are called *enclaves*, and loads the code into them. The enclaves are designed to run code in a trustworthy manner, even if the operation system is untrusted. The enclaves can achieve three functions:

- Isolation: Aims to put the data and program into the enclave; they can not be read or modified by any other external process.
- Sealing: Every SGX processor has a hardware-resident key which is called the Root Seal Key. When an enclave is created, it can derive a key from the Root Seal Key called the Seal Key, and the key is used to encrypt or authenticate data and store it in untrusted memory. Sealed data can be recovered by the same enclave on the same platform even after the enclave has been destroyed. However, the Seal Key cannot derived from different enclaves on the same platform or enclaves on a different platform.
- Attestation: There are two forms of attestation: *local* and *remote*. Local attestation is between two enclaves on the same platform. They can derive a shared key for authentication because they share the same Root Seal Key. Remote attestation is to generate a report that can be verified by a remote party.

Based on the functions of SGX, it is applied to provide a trusted computation environment in the proposed scheme to compute the duplicate blocks between two frames in a video. Thus, the proposed system is restricted based on the Intel service.

## 3. Problem Formulation

### 3.1. The Entities

Regarding the system model, three entities are discussed, including the context provider, the remote server and the transmission channel.

- Context provider: The entities which collect data and transmit them with other end-terminals or the remote server on the Internet host. The common context providers include the sensor node, RFID reader and so on. In most cases, the end terminals in IoT are limited in terms of both storage and computation resources.
- Remote server: The entity which hosts with a large capability of data storage and computational power. The users can store their data on the remote server and exploit its capability to accomplish computational tasks with abundant resources.
- Transmission channel: The entity which is set up to exchange data between the terminals or between the terminal and the remote server. In IoT, the transmission channels are usually public with barely any secure protection, such as the wireless channels.

### 3.2. System Goals

In this paper, we discuss the duplicate removal storage system in the IoT, which provides efficient centralized management of the data collected by the terminals. The challenge is the realization of private-preserving deduplication on the remote server side. Privacy preservation implies that the computational task should be conducted in the encrypted form. So, the proposed system aims to reduce the required storage space via a deduplication scheme as well as protect personal privacy via an encryption mechanism. The system goals can be elaborated in terms of these two aspects.

#### 3.2.1. Deduplication

Recently, the storage of data has become more and more centralized due to the increase in bandwidth over the Internet. In the cloud-based IoT scenario, the large amount of data collected by context providers is transmitted to the remote server in the cloud side.

It is envisioned that the volume of digital resources produced in IoT will increase at an alarming rate. Instead of expanding the space of data storage endlessly, it has been noted that a high redundancy of data makes inefficient use of storage resources on the server. Therefore, data deduplication has been widely adopted to allow effective utilization of storage space. The prospects for storage optimization via deduplication are promising. If a storage system has the ability to delete duplicate data, people simply call it a duplicate removal storage system. In this paper, we propose a private-preserving version of deduplication which is conducted in videos in the encrypted form. Note that, for typical multimedia, videos are big in size and have a high redundancy.

#### 3.2.2. Privacy Preservation

The development of IoT has raised important concerns regarding the security of user-related content. In real-world applications of IoT, the focus of secure storage systems is on the protection of users’ privacy towards adversaries. The security threats come from two main aspects. First, most communication channels in IoT are public, such as wireless channels, which makes eavesdropping quite simple. Once the outside adversaries grab the data, user privacy is aggrieved. Second, the remote servers are always considered to be the curious-but-honest third parties, which are outside of users’ trusted domains. For their own benefits, those curious-but-honest parties may attempt to learn some privacy information about clients from the data while following protocol steps to complete the storage or computation tasks.

Due to these concerns, the context providers should avoid transferring private data which may reveal their personal information such as the position, preference or environment to the remote server without protection. Considering that cryptosystems have been widely adopted for data protection, in the current study, we try to solve the private-preserving deduplication problem based on the prevalent encryption methods.

## 4. System Implementation

In the last section, the system’s goal was described as the realization of a centralized duplicate removal storage system with privacy preservation in IoT. This paper targets the deduplication of typical multimedia data such as images and videos. This section will describe the detailed implementation of a privacy-preserving duplicate removal storage model in IoT.

For the purpose of effective deduplication, the properties of multimedia data provided by the end-terminals in IoT are firstly analyzed. Multimedia data usually have intrinsic features, such as a high redundancy and a high level of correlation. Moreover, the collections of multimedia content by a particular terminal in IoT are often carried out in fixed environments, such as a user’s working site and home. This means that the collected multimedia content must contain lots of duplicate content.

In common IoT situations, if the data center has the ability to eliminate the redundancy of the collected multimedia data, the storage space can be greatly reduced. This problem of deduplication on text files has been already well studied [26,41,42]. However, there has been less focus on the deduplication of multimedia data which is more complicated. In addition, previous studies about data deduplication did not consider the problem of private preservation, which is an indispensable part of a secure duplicate removal storage system in IoT.

The basic deduplication methods generally fall into two categorical types based on different granularities: file-level deduplication and block-level deduplication. For each type, the private-preserving the realization of image deduplication is proposed in the encrypted form. Compared to the original deduplication methods, the proposed duplicate removal storage system takes both storage saving and privacy protection into consideration. The operation of data deduplication is transferred to the encrypted form for privacy preservation.

### 4.1. File-Level Deduplication System with Privacy Preservation

**Figure 1:**
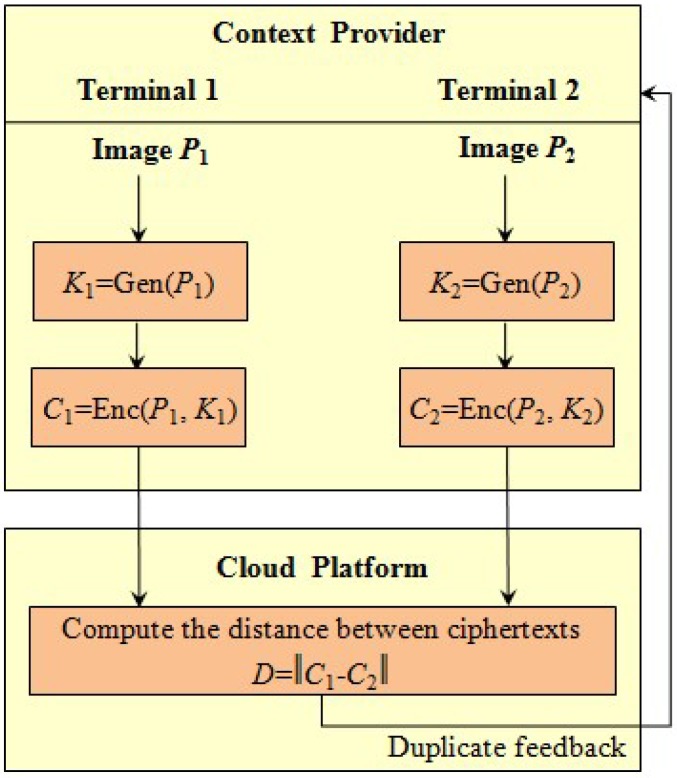
The mechanism of private-preserving file-level deduplication system based on the convergent encryption.

In file-level deduplication, the files are identified as duplicated copies if every pixel of exactly matches. Using this context, the operation of pixel comparison with privacy preservation based on the convergent encryption is achieved. As illustrated in Figure 1, the deduplication system involves the context providers and the cloud platform in IoT scenarios. In the terminal sides, the collection of contexts is conducted. Meanwhile, the terminals take charge of image encryption before transmission. In the cloud platform, the obtained ciphertexts are stored in the server and the deduplication is conducted in the encrypted form. The following steps describe the procedures of the privacy-preserving file-level deduplication scheme.

Step 1: The image, *P*, collected by the terminal is firstly used to calculate the convergent key, *K*, as required in the convergent encryption:

(5) K=Gen(P).

Step 2: The ciphertext is produced by encrypting the plain image, *P*, with the key *K*:

(6) C=Enc(P,K).

Step 3: The terminal sends the ciphertext, *C*, to the cloud platform through a public transmission channel. Then the local copy can be deleted.

Step 4: In the cloud platform, the server compared ciphertexts in the file-level deduplication which requires complete matching. The following equation is used to present the ciphertexts exactly matches:

(7) $$∥C_{1}-C_{2}∥=0.$$

From the property of convergent encryption, it can be easily inferred from identical ciphertexts that the corresponding plaintexts are identical copies.

Step 5: When the duplicated copies are determined as identified copies by the server, the server feeds back this result to the terminals. Meanwhile, the duplicated ciphertext is removed on the storage server in the cloud platform. In this context, only a unique copy is finally reserved instead of keeping multiple image copies in the cloud side. In addition, all of the deleted images are replaced with a pointer to the unique copy twhich is stored on the server.

### 4.2. Block-Level Deduplication System with Privacy Preservation

As described in Section 4.1, as the file-level deduplication system with privacy preservation system is able to duplicate only identical copies. Thus, the reduction of storage by this deduplication method is limited. Therefore, block-level deduplication is adopted to allow a greater saving of storage space than that of file-level deduplication. This section focuses on the framework for video deduplication.

A video consists of several consecutive sequences of frames. It is well known that neighboring video frames are strongly correlated to each other, which means that a high level of redundancy exists in video files. Storage of this high redundant data makes inefficient use of storage resources. Since the frames are not completely identical, but are strong correlated and locally similar, file-level deduplication may not be appropriate in this case. If the redundancy of video frames can be eliminated, the space required for storing the videos will be significantly reduced.

The performance of block-level deduplication on the granularity of blocks system has been studied in recent years [25,26,31]. However, the private-preserving block-level deduplication of multimedia data, which takes both storage and security performance into consideration, is still largely ignored. In the proposed block-level deduplication system, deduplication is not only conducted on the blocks of a single image, but also on the blocks of different images in order to improve the efficiency of deduplication further. In this context, privacy-preserving block-level deduplication is proposed in the following section.

#### 4.2.1. Block-Level Deduplication System Based on Convergent Encryption

**Figure 2:**
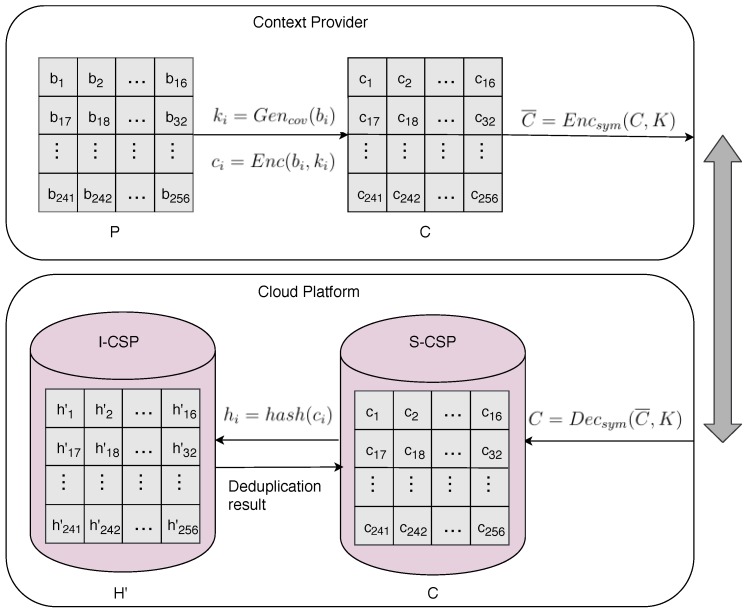
The block-level deduplication system based on the convergent encryption framework.

In this subsection, a block-level deduplication system based on the convergent encryption is described. The system aims to achieve two main goals. On one hand, the deduplication work will be conducted on blocks of images, which can reach a much higher deduplication ratio. On the other hand, to protect users’ private information, the data must be encrypted before being transmitted onto public channels to avoid eavesdropping. Meanwhile, the original information is unknown to the data center if the task of deduplication is finished in the encrypted form. Figure 2 shows the block-level deduplication system.

The main steps of the block-level deduplication system in the terminal side are described as follows.

Step 1: The content of image *P* collected by the terminals is divided into *n* blocks, and $b_{i}$ denotes the $i^{th}$ block of image *P*:

(8) $$P=\{b_{1},b_{2},\ldots ,b_{n}\}.$$

Step 2: The block, $b_{i}$, is used to compute the convergent key:

(9) $$k_{i}=Gen_{cov}\left(b_{i}\right)\;i=1,2,\ldots ,n.$$

Step 3: The terminal encrypts every block with the corresponding $k_{i}$:

(10) $$c_{i}=Enc\left(b_{i},k_{i}\right)\;i=1,2,\ldots ,n.$$

Thus, $C=\{c_{1},c_{2},\ldots ,c_{n}\}$ is the result of convergent encryption on *P*. Note that the size of *C* is the same as *P*.

Step 4: In the second round of encryption, the ciphertext, *C*, is encrypted by the symmetric key, *K*, which is shared between the terminal and the cloud platform at the beginning of communication:

(11) $$\bar{C}=Enc_{sym}\left(C,K\right).$$

Then, the terminal transmits the ciphertext, $\bar{C}$, to the cloud platform through the public channel.

In the cloud side, as the server receives the ciphertext, the block-level image deduplication in the encrypted form is conducted by the following steps.

Step 5: The ciphertext is decrypted with the symmetric key, *K*, on the server:

(12) $$C=Dec_{sym}\left(\bar{C},K\right).$$

The server obtains $C=\{c_{1},c_{2},\ldots ,c_{n}\}$, the result of convergent encryption on each block of images, which are stored on the Storage-Cloud Service Provider (S-CSP).

Step 6: To speed up the deduplication conducted in the encrypted form, the vector of blocks $C=\{c_{1},c_{2},\ldots ,c_{n}\}$ are mapped to the vectors of hash values $H=\{h_{1},h_{2},\ldots ,h_{n}\}$ by a hash function:

(13) $$h_{i}=hash\left(c_{i}\right)\;i=1,2,\ldots ,n.$$

Then, $H=\{h_{1},h_{2},\ldots ,h_{n}\}$ is stored on the server’s Index-Cloud Service Provider (I-CSP).

Step 7: Suppose there is a similar image, $H^{\prime }=\left\{h_{1}^{\prime },h_{2}^{\prime },\ldots ,h_{n}^{\prime }\right\}$, in which $h_{j}^{\prime }$ denotes the $j^{th}$ block. The elements are compared between $H^{\prime }$ and *H*. After deleting the duplicated elements, the no-duplicate vectors, $H_{NoDup}^{\prime }=\left\{h_{j_{1}}^{\prime },h_{j_{2}}^{\prime },\ldots ,h_{j_{l}}^{\prime }\right\}$, are obtained, satisfying

(14) $$h_{i}^{\prime }\neq h_{i},\;ifi=j\;i,j=1,2,\ldots ,n.$$

Then, I-SCP feeds back the result of the duplicated subscripts to S-CSP. So, S-CSP can deleted the duplicated blocks in C and obtain a no-duplicate vector of cipher blocks, denoted as $C_{NoDup}=\left\{c_{i_{1}},c_{i_{2}},\ldots ,c_{i_{n}}\right\}$.

**Figure 3:**
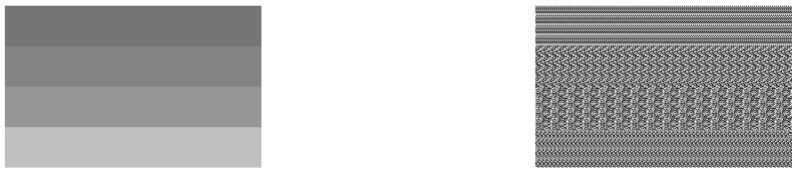
The similar statistical characteristics in the original image and block-based cipher image using convergent encryption.

The block-level deduplication system described above is not secure because of the leakage of statistical information. By making use of convergent encryption, the server is able to perform deduplication in the encrypted form. However, if the convergent encryption is directly applied to each block of images in the file-level deduplication, this will lead to the leakage of statistical characteristics in the original images. The reason for this is that the same result is obtained from the same block by convergent encryption. Therefore, the eavesdropper over the public channel will learn a lot of statistical information from the ciphertext. Figure 3 shows the encrypted results of an image, block by block, using convergent encryption. It is not hard to find the blocks of the original image and the cipher image repeats in the same way, which verifies the concerns about the leakage of statistical information.

In order to avoid such information leakage, we considered exploiting a second round of encryption on the convergent encryption results to disturb the statistical property. Thus, the symmetric encryption scheme was adopted to disturb the statistical characteristics. However, the second round of encryption brings a challenge during the removal of duplicate blocks. In the above scheme, the server needs to firstly decrypt the ciphertext, $\bar{C}$, before comparing processes. The server is honest-but-curious in the assumption, which means that it may try to obtain the statistical information after decryption. Thus, it is considered insecure.For the above reasons, a secure block-level deduplication system based on convergent encryption using the SGX technique [40] is proposed.

#### 4.2.2. Secure Block-Level Deduplication System Using SGX

**Figure 4:**
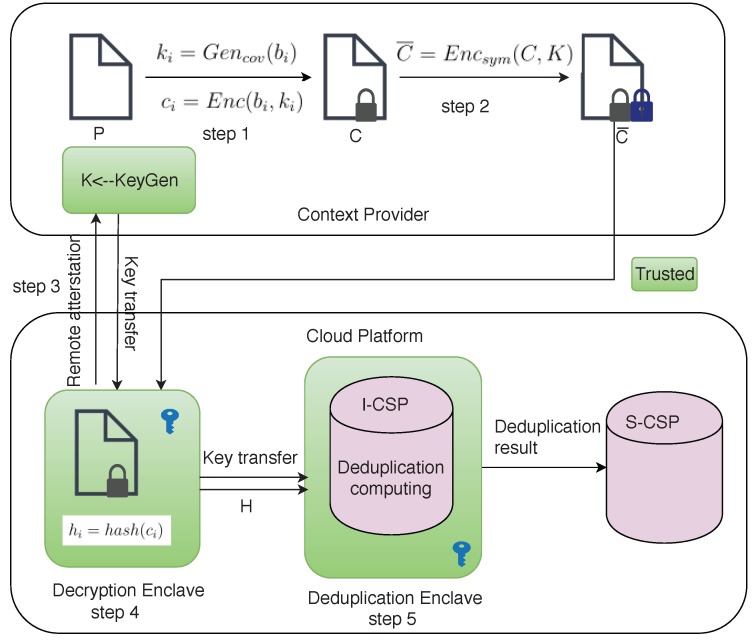
The secure block-level deduplication system based on convergent encryption using SGX.

In this subsection, the SGX technique is utilized to achieve the block-level deduplication system based on convergent encryption, which is illustrated in Figure 4. Because of the three advantages of SGX, *isolation, sealing* and *attestation*, the process of deduplication removal is transferred to the enclaves which are secure, isolated execution environments. Consequently, it is hard for the server to obtain the statistical information when it performs deduplication.

The secure block-level deduplication system includes the terminal side and the server side. However, both sides need to interact. The main steps are similar to the above description in Section 4.2.1.

Step 1: The content in image *P* collected by the terminals is divided to *n* blocks, and $b_{i}$ is denoted as the $i^{th}$ block of image *P*, e.g., $P=\{b_{1},b_{2},\ldots ,b_{n}\}$. The block, $b_{i}$, is used to compute the convergent key:

(15) $$k_{i}=Gen_{cov}\left(b_{i}\right)\;i=1,2,\ldots ,n.$$

Then, the terminal encrypts each block, $b_{i}$, with the corresponding convergent key, $k_{i}$:

(16) $$c_{i}=Enc\left(b_{i},k_{i}\right)\;i=1,2,\ldots ,n.$$

Thus, $C=\{c_{1},c_{2},\ldots ,c_{n}\}$ is the result of convergent encryption on *P*. Note that the size of *C* is the same as *P*.

Step 2: In order to avoid an eavesdropper intercepting information when the content provider sends messages to the cloud server, a symmetric key, *K*, is used to encrypt the information in the second round of encryption:

(17) $$\bar{C}=Enc_{sym}\left(C,K\right).$$

Step 3: When the decryption enclave is initialized, it firstly performs remote attestation on the content provider. If the attestation is valid, then a secure channel is established, and the decryption enclave receives the secret key, *K*, over this secure channel. The content provider loads the ciphertext, $\bar{C}$, into the decryption enclave.

Step 4: In the decryption enclave, the program runs as follows:

(18) $$C=Dec_{sym}\left(\bar{C},K\right).$$

After decryption, the vectors of blocks $C=\{c_{1},c_{2},\ldots ,c_{n}\}$ is mapped to the vectors of hash values $H=\{h_{1},h_{2},\ldots ,h_{n}\}$ by a hash function that is used to speed up the deduplication conducted:

(19) $$h_{i}=hash\left(c_{i}\right)\;i=1,2,\ldots ,n.$$

Step 5: The deduplication enclave receives the secret key, *K*, and hash values, *H*, from the decryption enclave through local attestation. In the deduplication enclave, the deduplication computing is conducted by Step 7 in Section 4.2.1. After deleting the duplicated elements, the I-CSP returns the results of the duplicated subscripts to the server S-CSP. So, S-CSP can deleted the duplicated blocks in *C* and obtain a no-duplicate vector of cipher blocks.

The security of the proposed system is ensured by the following two aspects. First, convergent encryption hides the content of the video, and the symmetric encryption protects the statistical information from eavesdropping during transfer. Thus, double encryption in the system is needed. Second, the SGX technique is utilized to protect the deduplication from leaking statistical information to the server provider. The security is based on the SGX because it can provide a secure, isolated environmen; programs and data inside the enclave cannot be read or modified by any other attackers.

## 5. System Evaluation

To verify that the proposed system is practical and it is available to duplicate centrally in IoT with privacy preservation, this section focuses on evaluating the performance with regard to (1) the ratio of deduplication; and (2) the comparison of storage space between the proposed scheme and video compression algorithms.

**Figure 5:**
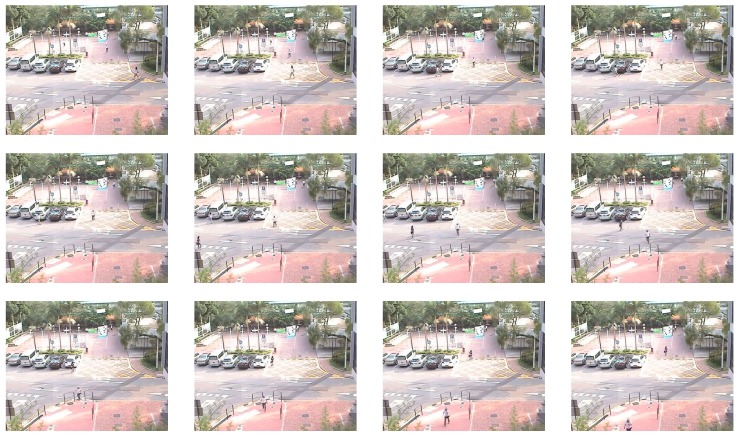
The adjacent frames extracted from a traffic video sequence, recorded by a stationary camera.

All of the experiments were performed on computer with Intel (R) Core(TM) i7-7500U CPU 2.7GHZ with 8G RAM. As shown in Figure 5, a series of continuous frames were extracted from a traffic video sequence recorded by a stationary camera, which is supported by [43]. Each frame was resized to 256×256 and divided into 256 blocks of size 16×16. The proposed system was implemented as described in Section 4, and its performance was evaluated.

**Figure 6:**
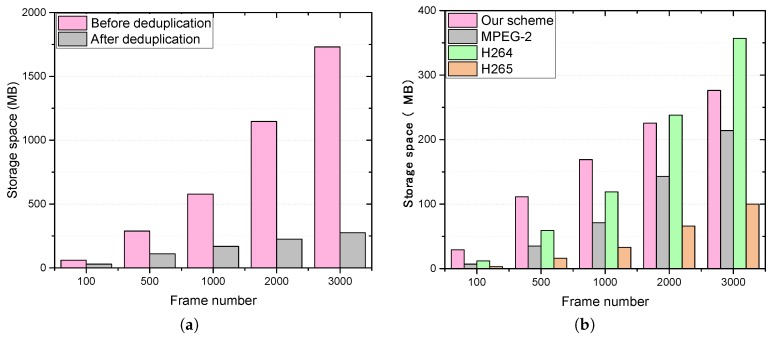
(**a**) Comparison of storage space between before and after removal in the proposed system; (**b**) comparison of storage space between the proposed system and compression algorithms.

As for the ratio of deduplication, the deduplication scheme was applied on the video and the results from 100 to 3000 frames are reported in Table 1. The ratio of deduplication increased to 84.04% when the number of frames reached 3000, which means that the overall compression ratio for the images yielded a space saving of 84.04%. So, the requirement for storage space for these 3000 frames was only about 16% of the required storage space before deduplication. Figure 6a illustrates the comparison of storage space between the original video and the video after deduplication. It can be seen that with an increase in frame number, the deduplication ratio increased rapidly; thus, the proposed system can greatly reduce storage space.

**Figure 7:**
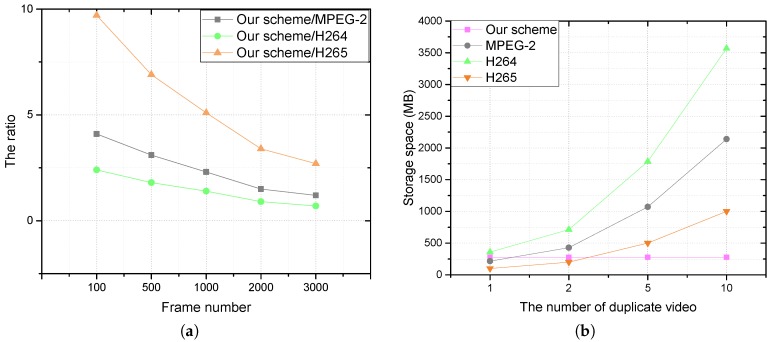
(**a**) The ratio of the proposed scheme to compression algorithms; (**b**) comparison of storage space between the proposed system and compression algorithms when a video is duplicated.

Video compression is the main method used to save storage space by removing redundancy in a video. There are two differences between our scheme and the existing video compression methods. First, the proposed scheme can remove the redundancy between different videos, while the common video compression methods only duplicate the redundant data in a video. Second, it solves the problem of privacy-preserving video deduplication performed in the encrypted form on the cloud side. In order to verify that the proposed scheme can effectively remove duplicate data with privacy protection, we compared the performance of this scheme with the popular video compression algorithms, such as MPEG-2, H264 and H265. As shown in Figure 6b, although the proposed scheme does not achieve the minimum storage space after deduplication, it is better than H264 when the video is large enough. Meanwhile, the deduplication ratio of the proposed scheme and compression algorithms decrease with an increase in frame number, as shown in Figure 7a. Furthermore, when there are multiple identical or similar videos in the cloud server, the proposed scheme has advantages over traditional video compression methods. Figure 7b illustrates that our scheme does achieve the best performance in this case.

Overall, the proposed system is practical and reduces the cost of content providers by outsourcing the computing and storage burden to cloud service. More importantly, it protects the privacy of users’ data from disclosure by implementing deduplication in the encrypted form.

## 6. Conclusions

In the IoT environment, since most end-terminals have limited capacity for storage and computing power, users usually outsource the data from local to cloud-based computing. As the amount of data collected by the terminals in IoT is increasing at a surprising rate, it is necessary that data deduplication is adopted in storage centers to reduce the communication bandwidth and storage space. In this article, a centralized privacy-preserving duplicate removal storage system was built, which supports both file-level and block-level video deduplication. In order to avoid the leakage of statistical information of data, SGX technology was utilized to protect the deduplication process on the cloud server. Experiments showed that the duplicated removal system performs efficiently with an enhanced level of security. It is envisioned that the duplicated removal system with privacy-preserving will be of great use in the centralized storage environment of IoT.

## Figures and Tables

**Table 1:** Deduplication ratio for the video using the duplicate removal storage system.

Frame number	100	500	1000	2000	3000
Block number	25,600	128,000	256,000	512,000	768,000
Duplicated number	13,101	78,875	181,108	411,352	645,410
Deduplication ratio	51%	61.61%	70.75%	80.34%	84.04%
Required storage space	49%	38.48%	29.25%	19.66%	15.96%
